# Cytomorphometric changes in the dorsal raphe neurons after rapid eye movement sleep deprivation are mediated by noradrenalin in rats

**DOI:** 10.1186/1744-9081-6-62

**Published:** 2010-10-21

**Authors:** Amit Ranjan, Sudipta Biswas, Birendra N Mallick

**Affiliations:** 1 School of Life Sciences, Jawaharlal Nehru University, New Delhi 110067, India; 2 Current address: Behavioral Neuroscience Division, Dept. of Psychology, Arizona State University, Tempe, AZ 85287-1104, USA

## Abstract

**Objectives:**

This study was carried out to investigate the effect of rapid eye movement sleep (REMS) deprivation (REMSD) on the cytomorphology of the dorsal raphe (DR) neurons and to evaluate the possible role of REMSD-induced increased noradrenalin (NA) in mediating such effects.

**Methods:**

Rats were REMS deprived by the flowerpot method; free moving normal home cage rats, large platform and post REMS-deprived recovered rats were used as controls. Further, to evaluate if the effects were induced by NA, separate sets of experimental rats were treated (i.p.) with α1-adrenoceptor antagonist, prazosin (PRZ). Histomorphometric analysis of DR neurons in stained brain sections were performed in experimental and control rats; neurons in inferior colliculus (IC) served as anatomical control.

**Results:**

The mean size of DR neurons was larger in REMSD group compared to controls, whereas, neurons in the recovered group of rats did not significantly differ than those in the control animals. Further, mean cell size in the post-REMSD PRZ-treated animals was comparable to those in the control groups. IC neurons were not affected by REMSD.

**Conclusions:**

REMS loss has been reported to impair several physiological, behavioral and cellular processes. The mean size of the DR neurons was larger in the REMS deprived group of rats than those in the control groups; however, in the REMS deprived and prazosin treated rats the size was comparable to the normal rats. These results showed that REMSD induced increase in DR neuronal size was mediated by NA acting on α1-adrenoceptor. The findings suggest that the sizes of DR neurons are sensitive to REMSD, which if not compensated could lead to neurodegeneration and associated disorders including memory loss and Alzheimer's disease.

## Background

Rapid eye movement sleep (REMS) is a unique but an essential physiological phenomenon expressed at least in higher-order mammals, including humans. REMS deprivation (REMSD) affects several psychosomatic illnesses and prolonged deprivation may lead to death [1,2]. It also causes several biochemical and behavioral changes [3,4]. At the cellular level REMSD has been reported to affect intracellular calcium levels [5], membrane fluidity [6], expressions of various proteins including several enzymes [3,7,8] and cytoskeletal proteins [7]. REMSD-induced changes in Na-K ATPase [9], which is primarily responsible for maintenance of ionic gradient across the cell membrane [10], and transmembrane potential of the neurons [11] have been studied systematically in relative detail. Changes in neuronal Na-K ATPase activity, calcium concentration and structural proteins are likely to affect neuronal morphology, integrity, functioning and life span. In support, we have reported cytomorphometric changes in noradrenalin (NA)-ergic, cholinergic and GABA-ergic neurons in locus coeruleus (LC), pedunculopontine region and medial preoptic area of rats after REMSD [12]. We have also observed evidence suggesting increased apoptosis, disintegration of cytoskeleton and loss of neurons in the above mentioned regions in the rat brain after REMSD, which may have relevance to REMS loss associated changes in higher integrated processes and diseases e.g. Alzheimer's disease [7,13,14].

NA is one of the key neurotransmitters involved in REMS regulation [14]; its level in the brain reduces during REMS [15] and increases during REMSD [3,16]. The NA-ergic neurons are predominantly concentrated in the LC of rats [17] and these neurons are primarily responsible for supplying most of the NA throughout the brain, including the dorsal raphe (DR), the main site for serotonergic neurons in the brain [18,19]. The DR neurons are reported to behave like the LC neurons, particularly in relation to REMS, which has been proposed to be due to the withdrawal (disfacilitation) of excitatory inputs from the LC neurons [20]. The DR neurons continue firing during REMS without atonia [21]. Further, since our previous studies have shown that REMSD-induced cytomorphometric changes in neurons were induced by NA [12] and that NA level increases in the brain after REMSD [16,22,23], we hypothesized that DR neurons also are likely to get affected by REMSD induced elevated levels of NA. Alzheimer's disease associated reduction in both REMS as well as serotonin level [24,25] supports our hypothesis. Therefore, in the present study, we evaluated cytomorphology of DR neurons in experimental REMS-deprived rats and compared them with various control as well as PRZ-treated rats. We observed that after REMSD the mean size of the DR neurons increased significantly as compared to that of the control rats and that the effects of REMSD were not observed in the PRZ-treated group, suggesting that the effect of REMSD were mediated by NA.

## Methods

Experiments were conducted on inbred male wistar rats (250--300 g) maintained in standard home cages under 12/12 h light/dark cycle (lights on 7:30 AM) with ad libitum access to food and water. The experiments were approved by the Institutional Animal Ethics Committee and every effort was made to minimize the number of animals used and their sufferings. Free- moving control (FMC) rats were maintained in their normal dry home cages. Experimental rats were REMS deprived by the classical flowerpot method for 6 days because under identical conditions REMSD for shorter period did not significantly affect the cytomorphology of the LC and other neurons [12]. For REMSD, individual rats were maintained on a 6.5-cm diameter platform surrounded by water. To rule out nonspecific effects, another group of control rats was maintained on large (13-cm diameter) platform (LPC) surrounded by water i.e. except for larger-platform diameter, all other conditions remained identical to that of the experimental animals. A fourth recovery (REC) group of rats included those animals which had been REMS deprived for 6 days and were then allowed to live in normal cages for 3 days to recover from lost REMS. In addition, in a separate set of experiment, PRZ (2 mg/kg body wt, Sigma-Aldrich, St Louis, MO) was intraperitoneally (i.p.) injected once a day to the rats during the last 4 days of REMSD; the dose was used based on our previous findings [12].

At the end of experiment, the controls as well as experimental rats were anesthetized with ketamine-xylazine (80 mg/kg and 32 mg/kg, respectively, i.p.; Chandra Bhagat Pharma Pvt. Ltd, India). The brains were intracardially perfused with 0.1 M phosphate buffer saline (PBS) and 4% paraformaldehyde in 0.1 M phosphate buffer (pH 7.4). The brains were fixed overnight in the same fixative and were cryoprotected in 30% sucrose in PBS. Thereafter, 40 μm frozen cryostat sections (Leica, Solms, Germany) through the antero-posterior extension of inferior colliculus (IC) and DR, as per the rat brain atlas of Paxinos and Watson [26], were collected in vials containing PBS and were stored at 4°C for staining. Alternate sections through DR and IC were taken onto gelatin-coated slides and were processed for Nissl staining. The sections on subbed slides were stained with 1% cresyl violet and 0.1% thionin in acetate buffer following standard protocol of Nissl staining as reported earlier [12]. The stained sections were dehydrated through increasing concentration (30%, 50% and 70%) of ethanol for 5 minutes each while for 1 minute each in 90% and 100% ethanol, cleared in xylene, cover slipped with distrene plasticizer xylene (DPX) and air dried before observing under microscope for cytomorphometric analysis. The slides were coded and analyzed by different researchers at random to minimize bias.

**Figure 1:**
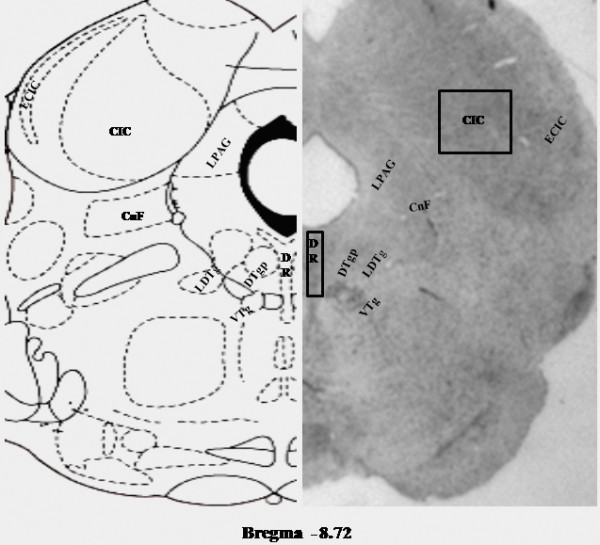
**Representative histological section through DR and IC (bregma -8.72 mm) on right half along with corresponding anatomical brain atlas (Paxinos and Watson, 1998) section on the left half are shown**. The neurons in areas marked as DR and CIC, respectively, were studied. Abbreviations: DR, dorsal raphe; CnF, Cuniform nu; CIC, central nu of inferior colliculus; DTg, dorsal tegmental nucleus; ECIC, external cx of inferior collic; LDTg, laterodorsal tegmentum nu; LPAG, lateral periaqueductal gray; VTg, ventral tegmental nu.

All the experiments were repeated in 4 sets and in each set there was one rat each of FMC, REMSD, LPC, REC and PRZ-treated. The histochemically stained sections were observed under Nikon E400 microscope (magnification 400X) and DR as well as IC was identified anatomically as marked on the atlas (Figure 1) [26]. The magnified views of neurons in these areas were captured in a computer with the help of a charged coupled device (CCD) camera (JVC, Tokyo, Japan) fitted to the microscope by using dedicated software Image-Pro plus 5.1.1 (Media Cybernetics, Silver Spring, MD).

Only the neurons where nuclear boundaries	were	visible	were	selected for cyto-morphometric analysis. Boundaries of 30 to 50 neuronal perikarya from every third serial section were traced on the computer screen (with the help of a computer mouse) and their perimeters as well as areas were estimated by using cytomorphometric analysis software (Image- Pro plus 5.1.1). Five to seven such sections were studied from each animal and there were 4 animals per treatment group. Thus on an average 900 ± 70 neurons were estimated from each anatomical brain areas and each treatment group (FMC, LPC, REMSD, REC and PRZ). Further, another coworker randomly reconfirmed the estimates in 3% to 5% of neurons.

Intra-group analysis of variance (ANOVA) and normality tests on the data from all the animals of the same group (FMC, LPC, REMSD, REC and PRZ) were conducted using Sigma Stat software (Jandel Scientific, USA). Since the values were statistically comparable, the data from all the animals of the same group were pooled for further statistical analysis. The inter- group ANOVA analysis between mean area and perimeter of the experimental (REMSD) and each of the control groups (FMC, LPC, REC and PRZ) were conducted using the same software. Similarly, data from within control groups were also compared using ANOVA. The mean area and mean perimeter were used to calculate the soma form factor (FF) using the equation, FF = 4π (soma area/perimeter^2^) [27] to evaluate the changes in the shape of the neurons.

## Results

Neurons occupy space in 3-Dimension (3-D); however, in histological sections under microscope, we observe them in 2-D space. Hence, in order to get the maximum information, we estimated the surface area as well as the perimeter of each neuron and reasonably extrapolated possible changes in the size and shape of those neurons caused by REMSD. The intra-group data from all the 4 animals within each of the 5 groups of rats were statistically comparable; the *p*- values for DR neuronal area and perimeter in FMC, LPC, REMSD, REC and PRZ groups were (0.38, 0.47, 0.43, 0.50 and 0.06) and (0.07, 0.10, 0.11, 0.64 and 0.42), respectively. Data in each group also passed normality test (*p *> 0.2), suggesting that the data followed a normal distribution and hence data from different animals in the same group were pooled for further statistical inter- group ANOVA analysis between mean area and perimeter of the experimental (REMSD) and each of control groups (FMC, LPC, REC, and PRZ).

### Cytomorphometric changes in the dorsal raphe neurons after various treatments

#### (1) Changes in the surface area

**Figure 2:**
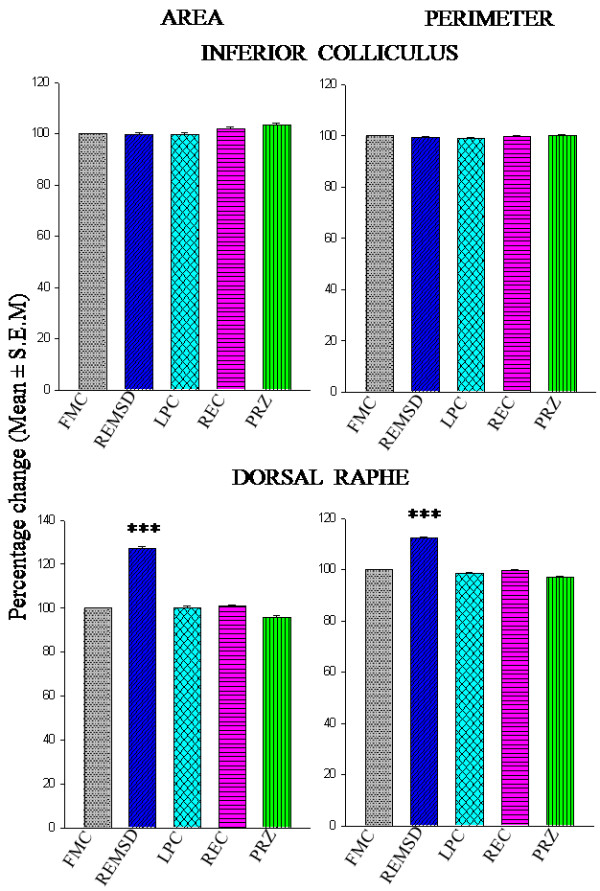
**Percentage changes in mean (± SEM) area and perimeter of neurons located in IC and DR in Nissl-stained sections under experimental (REMSD), treated (PRZ), and control (LPC and REC) conditions as compared to that of FMC taken as 100% are represented by histograms**. Abbreviations are same as in the text, ***P < 0.001 significantly different from FMC.

**Figure 3:**
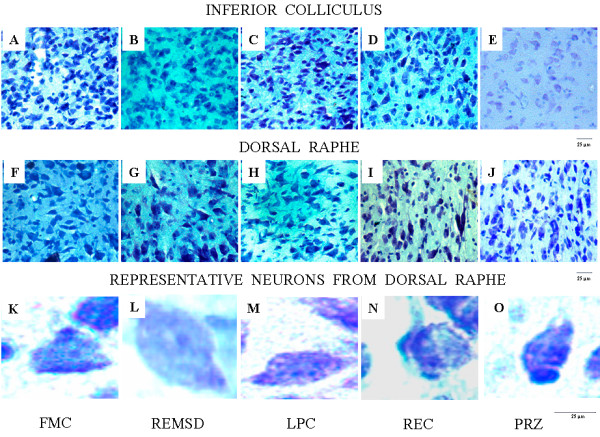
**Representative photomicrographs of Nissl-stained sections under different experimental and control conditions are shown in this figure**. Neurons in IC (upper panel; A, B, C, D and E) and DR (middle panel; F, G, H, I and J) are shown under different conditions. Magnified representative single cell in DR under different conditions are shown in (lower panel; K, L, M, N and O). All the photomicrographs from (upper and middle panels) are at 400X magnifications, whereas those of single neurons at (lower panel) are under 1000X magnification. Scale bars = 25 μm. Abbreviations are same as in the text.

The mean (± SEM) surface area of Nissl-stained DR neurons in FMC was 122.13 ± 1.03 μm^2^, which increased to 155.15 ± 1.38 μm^2^ after REMSD; an increase of 127.04% and was statistically significant [F (1,7) = 367.69, *p *< 0.001] as compared to FMC. The mean (± SEM) surface area in LPC, REC and PRZ groups of rats were 122.30 ± 0.98 μm^2^, 123.19 ± 0.88 μm^2^, 117.06 ± 0.80 μm^2^, which were comparable to that of the FMC and among each other. The mean surface area of the neurons was not affected in control rats; however, the REMSD-induced increase in neuronal size was not observed in the REMS-deprived PRZ-treated (Figure 2 and Figure 3).

#### (2) Changes in the perimeter

The mean (**± **SEM) perimeter of DR neurons increased from 49.54 ± 0.22 μm in FMC to 55.64 ± 0.26 μm in REMSD; an increase of 112.31% [F (1,7) = 320.776, *p *< 0.001] as compared to FMC. The mean perimeter of the neurons in LPC, REC and PRZ treated rats was 48.91 ± 0.21 μm, 49.47 ± 0.19 μm and 48.15 ± 0.18 μm, respectively and they were statistically comparable to that of FMC as well as among each other. Thus, although REMSD increased the neuronal perimeter as compared to controls, the effect was not seen in REMS deprived rats treated with PRZ (Figure 2 and Figure 3).

#### (3) No changes in the soma form factor

The mean FF (± SEM) values of neurons in FMC (0.61 ± 0.02), LPC (0.64 ± 0.01), REMSD (0.62 ± 0.01), REC (0.63 ± 0.01) and PRZ (0.63 ± 0.01) treated rats were not significantly different. Since FF values were comparable, it indicated that REMSD-induced increase in the area of the neuronal perikarya was proportional to the increase in square of perimeter of the neurons in respective cases.

### Cytomorphometric changes in the inferior colliculus neurons after various treatments

The intra-group data from IC neurons also passed normality test (*p *> 0.2) and hence, they were pooled. The inter-group mean (± SEM) areas as well as perimeter (± SEM) of the IC neurons in FMC, LPC, REMSD, REC and PRZ-treated groups were not statistically different (Figure 2, Figure 3). The FF values were also statistically comparable among all the five groups.

## Discussion

In this study we observed that after REMSD the sizes of DR neurons increased in experimental rats as compared to that of the control rats. The effects were specific to REMSD and not non-specific effects because i) the neurons remained unaffected in LPC, where all other conditions remained identical as those of the experimental group; ii) the REMSD induced changes in neuronal size returned to the reference level (the FMC group) after recovery from REMSD (the REC group of rats); and iii) the affected neurons were located within the anatomical region of the brain, the DR in this case, where functional REM-OFF neurons have been reported [28,29]. This view may be supported by the fact that adjacent neurons in IC, where such REMS-related neurons have not been reported, remained unaffected. In our previous studies [12] rats were REMS deprived for 4, 6 and 10 days; significant changes were observed in the brain areas including the LC after 6 and 10 days of REMSD, whereas 4 days of REMSD was ineffective. To prevent unnecessary discomfort to the animals and to follow a uniform pattern of deprivation in this study also we used 6 days of REMSD paradigm.

To avoid estimating the same neuron more than once we analyzed every third serial section. Since the sections were 40 μ m thick it is unlikely that a neuronal soma would span three sections. Also, we selected neurons where the nuclear boundaries were clearly visible. The sampling error was minimized by counting a large number of neurons (900 ± 70) at random across each of the anatomical locations from 4 rat brains in each group. The intra-group data were not significantly different and hence the values were pooled for further analysis.

The flowerpot method [30] is reliable and it has been reported that when continued beyond 24 hrs it deprives animals maximally of REMS whereas non-REMS is least affected [31]; therefore, by far this is the best available method which has been extensively used globally for experimental REMSD studies. Notwithstanding, like most other experimental procedures this method also has some limitations, which are taken care of by using various control groups. A common criticism of the use of flowerpot method is that the animal may experience stress due to enforced immobilization and social isolation. Our previous studies have shown that stress, if at all, induced by restricted movement or muscular over-activity, by either keeping them in restricted space or making the rats swim for various lengths of time, respectively, were not the causes of flowerpot method induced REMSD-associated increase in Na-K ATPase and chloride-sensitive Mg-ATPase activities [9,32]. In some studies multiple platforms have been used to avoid possible social isolation [33], however, in our studies before the experiments, normally the rats were acclimatized for a few days by maintaining them individually in cages; hence, isolation is unlikely to have influenced the results. We have conducted LPC to rule out non-specific effects. In one study large platform rats also showed elevated level of plasma corticosterone [33]. However, in our studies since LPC rats did not show significantly increased Na-K ATPase and chloride- sensitive Mg-ATPase activities [9,32] nor did they show significant changes in neuronal sizes (neither earlier [12] nor in this study), it may be safe to say that changes observed by us in the experimental rats are most likely due to REMSD. Notwithstanding, it is also important to keep in mind that whichever method we use, in principle, experimental animals are subjected to a new environment, which by itself, may have some bearing on the results [34]; designing suitable control experiment is always a challenge to the experimenter. We are also aware of the other limitation of this study e.g. the same neuron could not be estimated before and after the treatment(s), as suffered by most cellulo-behavioral studies; hence, for confirmation one needs to study the same neuron before and after REMSD.

It was observed in this study that following REMSD, the neurons in DR increased in size as compared to controls; whereas neurons in IC were not affected. It has been reported that REMSD alters membrane fluidity [6], neuronal excitability [4,22], cytomorphometric changes [12] and neuronal death [7]. The increased excitability after REMSD is associated with increased Na-K ATPase activity [9], which among other functions, maintains the transmembrane potential and the osmotic balance within cells to stabilize cell volume [12,35]. On the other hand, studies have shown correlation between neuronal size and neuronal excitability [35,36]. Although it might offer some explanation for observing increased neuronal size in the DR after REMSD, it does not explain the possible mechanism of action and why IC neurons were not affected by the REMSD.

There is evidence that suggests the presence of REM-OFF type of cells in the DR nucleus [28] and those neurons do not cease firing during REMS without atonia [21]. It has been reported that the LC possesses REM-OFF as well as non-REM-OFF neurons and the former do not cease firing but instead continue firing during REMSD [37]. Since DR neurons apparently behave like REM-OFF neurons in relation to REMS, it is expected that those neurons might continue firing during REMSD. Tonic firing of neurons has been proposed to build up a significant metabolic and/or ionic debt [35]. Therefore, it is possible that upon REMSD an increased activity in DR neurons will cause increased Na^+^ concentration inside the neurons. REMSD-induced increased intracellular positivity, a reflection of depolarization of neurons, supports this view [38]. Increased Na^+^ concentration and metabolites inside a cell would cause increased water influx into the neurons due to osmosis, thus resulting in swelling and increased cell size [4,39]. However, recently it has been shown that a small fraction of DR neurons may not behave as REM-OFF type [40] and the DR contains serotonergic as well as non-serotonergic neurons [41]. Therefore, as a caution, it must be emphasized that all the DR neurons may not behave identically and such cellular and physiological differences cannot be distinguished from the results of this study.

We observed that a REMSD-induced increase in neuronal size was prevented by PRZ, suggesting that NA acting on α1-adrenoceptor was responsible for REMSD-induced increase in DR neuronal size. Although there is no direct evidence to suggest that NA increases DR neuronal size, the following circumstantial evidences support our contention; i) the LC NA-ergic neurons project to DR neurons and regulate their activity [18]; ii) the activity of DR neurons is turned off by the withdrawal of inputs from the LC during REMS [20]; iii) cessation of firing of the LC- NA-ergic neurons is a necessity for occurrence and maintenance of REMS [42]; iv) the NA-ergic neurons in LC cease activity during REMS but are continuously active during REMSD [37]; v) the NA-synthetic machinery are stimulated after REMSD [23]. Therefore, it is likely that since after REMSD the NA level increases, it in turn modulates the DR neuronal size by acting on α1- adrenoceptors. Our contention may be supported by the fact that REMSD did not affect the IC neurons, which do not possess α1- adrenoceptors [43]. Our earlier findings that REMSD increased Na-K ATPase activity [44,45] as well as size of LC-NA-ergic neurons [12] in the rat brain and both the effects were due to increased NA acting on α1- adrenoceptor further support the findings of this study. However, it needs to be investigated if the REMSD induced increased NA would have differential effects on neurons releasing different types of neurotransmitters because after REMSD although the size of the NA-ergic neurons increased, the size of cholinergic neurons showed a decrease [12].

The soma FF of neurons was calculated as reported earlier; neurons having FF value closer to 1 would be relatively rounder in shape than those closer to zero [27]. It is interesting to note that, in DR, although both the neuronal area and perimeter increased after REMSD, the FF values remained unaltered. This may happen only if the increase in area was proportional to the increase in square of perimeter. Such a condition is most likely to take place in case of smooth and regular-shaped neurons as compared to rough (undulated)-surfaced neurons. This observation is in sharp contrast to the effect of REMSD on LC neurons, where the FF values decreased [12]. It has been reported that morphologically neuronal soma in DR are smooth without any spines on the cell bodies [46], whereas the perikarya of many LC neurons possess many somatic protrusions, appendages or spines which render irregular surface to those neurons [47,48]. Hence, it is most likely that after REMSD, the rough or irregular surfaces of the LC neurons stretched or unfolded to become smoother and therefore, the increase in surface area of LC neurons was not proportional to the increase of square of the perimeter, whereas smoother surfaced DR neurons swelled proportionately. Subject to experimental confirmation we propose that DR neurons would be more vulnerable to damage and lysis after REMSD than that of LC neurons.

It has been reported that alterations in the size and shape of cells precede cellular degeneration [49]. It may be argued that neurons upon exposure to conditions detrimental to their survival try to compensate by altering sizes; however, in case of extreme insults, the compensatory mechanism(s) fail and the neurons undergo degeneration. Our finding suggests that neurons in DR increase in size after REMSD, which if not compensated could lead to neurodegeneration and contribute to memory loss, tremors, aggression, depression, psychosis and possibly increase the risk for neurodegenerative conditions, such as Alzheimer's disease. The findings of this study may be relevant to at least Alzheimer's disease because reduced levels of serotonin and imbalance between the serotonergic-cholinergic systems has been reported in patients with Alzheimer's disease [13] where REMS is also reduced [25]. These findings along with our previous results [7,12] show significant consequences of REMSD on public health, especially for nightshift workers, viz. nurses, frequent fliers to various time zones, BPO employees, truck drivers and others such as students and elderly people in whom sleep is significantly compromised.

## Competing interests

The authors declare that they have no competing interests.

## Authors' contributions

AR collected and analyzed the data as well as participated in preparing this MS; SB extended help while analyzing the data; BNM planned the study, arranged funds, trained co-workers and wrote the MS. All authors have read and approved the final manuscript.

## References

[B1] Mazet P Insomnia in infants: a frequent and early psychosomatic disorder Rev Neuropsychiatr Infant 1972 20 839 847 4650070

[B2] Rechtschaffen A Bergmann BM Everson CA Kushida CA Gilliland MA Sleep deprivation in the rat: X. Integration and discussion of the findings Sleep 1989 12 68 87 2648533

[B3] Mallick BN Madan V Faisal M Kushida CA Biochemical Changes: Sleep Deprivation 2005 192 New York: Marcel and Dekker 339 358

[B4] McDermott CM LaHoste GJ Chen C Musto A Bazan NG Magee JC Sleep deprivation causes behavioral, synaptic, and membrane excitability alterations in hippocampal neurons J Neurosci 2003 23 9687 9695 14573548 10.1523/JNEUROSCI.23-29-09687.2003 PMC6740462

[B5] Mallick BN Gulyani S Alterations in synaptosomal calcium concentrations after rapid eye movement sleep deprivation in rats Neuroscience 1996 75 729 736 10.1016/0306-4522(96)00177-7 8951869

[B6] Mallick BN Thakkar M Gangabhagirathi R Rapid eye movement sleep deprivation decreases membrane fluidity in the rat brain Neurosci Res 1995 22 117 122 10.1016/0168-0102(95)93696-Y 7792076

[B7] Biswas S Mishra P Mallick BN Increased apoptosis in rat brain after rapid eye movement sleep loss Neuroscience 2006 142 315 331 10.1016/j.neuroscience.2006.06.026 16887278

[B8] Majumdar S Mallick BN Increased levels of tyrosine hydroxylase and glutamic acid decarboxylase in locus coeruleus neurons after rapid eye movement sleep deprivation in rats Neurosci Lett 2003 338 193 196 10.1016/S0304-3940(02)01404-0 12581829

[B9] Gulyani S Mallick BN Effect of rapid eye movement sleep deprivation on rat brain Na-K ATPase activity J Sleep Res 1993 2 45 50 10.1111/j.1365-2869.1993.tb00060.x 10607070

[B10] Alberts B Bray D Lewis J Raff M Roberts K Watson JD Membrane transport of small molecules and ionic basis of membrane excitability: Molecular biology of the cell 1994 New York: Garland publishing 507 549 chap 11

[B11] Trachtenberg MC Packey DJ Sweeney T In vivo functioning of the Na+, K+-activated ATPase Curr Top Cell Regul 1981 19 159 217 6277572 10.1016/b978-0-12-152819-5.50022-1

[B12] Majumdar S Mallick BN Cytomorphometric changes in rat brain neurons after rapid eye movement sleep deprivation Neuroscience 2005 135 679 690 10.1016/j.neuroscience.2005.06.085 16154283

[B13] Garcia-Alloza M Gil-Bea FJ Diez-Ariza M Chen CP Francis PT Lasheras B Ramirez MJ Cholinergic-serotonergic imbalance contributes to cognitive and behavioral symptoms in Alzheimer's disease Neuropsychologia 2005 43 442 449 10.1016/j.neuropsychologia.2004.06.007 15707619

[B14] Gottesmann C Noradrenaline involvement in basic and higher integrated REM sleep processes Prog Neurobiol 2008 85 237 272 10.1016/j.pneurobio.2008.04.002 18514380

[B15] Shouse MN Staba RJ Saquib SF Farber PR Monoamines and sleep: microdialysis findings in pons and amygdala Brain Res 2000 860 181 189 10.1016/S0006-8993(00)02013-8 10727641

[B16] Mallick BN Singh S Singh A Mechanism of noradrenaline-induced stimulation of Na-K ATPase activity in the rat brain: implications on REM sleep deprivation-induced increase in brain excitability Mol Cell Biochem 2010 336 3 16 10.1007/s11010-009-0260-9 19823772

[B17] Aston-Jones G Bloom FE Activity of norepinephrine-containing locus coeruleus neurons in behaving rats anticipates fluctuations in the sleep-waking cycle J Neurosci 1981 1 876 886 7346592 10.1523/JNEUROSCI.01-08-00876.1981 PMC6564235

[B18] Jones BE Moore RY Ascending projections of the locus coeruleus in the rat. II. Autoradiographic study Brain Res 1977 127 25 53 301051

[B19] O'Leary OF Bechtholt AJ Crowley JJ Valentino RJ Lucki I The role of noradrenergic tone in the dorsal raphe nucleus of the mouse in the acute behavioral effects of antidepressant drugs Eur Neuropsychopharmacol 2007 17 215 226 10.1016/j.euroneuro.2006.06.012 16997535

[B20] Sakai K Crochet S Serotonergic dorsal raphe neurons cease firing by disfacilitation during paradoxical sleep Neuroreport 2000 11 3237 3241 10.1097/00001756-200009280-00037 11043555

[B21] Trulson ME Jacobs BL Morrison AR Raphe unit activity during REM sleep in normal cats and in pontine lesioned cats displaying REM sleep without atonia Brain Res 1981 226 75 91 10.1016/0006-8993(81)91084-2 7296301

[B22] Mallick BN Majumdar S Faisal M Yadav V Madan V Pal D Role of norepinephrine in the regulation of rapid eye movement sleep J Biosci 2002 27 539 551 10.1007/BF02705052 12381879

[B23] Porkka-Heiskanen T Smith SE Taira T Urban JH Levine JE Turek FW Stenberg D Noradrenergic activity in rat brain during rapid eye movement sleep deprivation and rebound sleep Am J Physiol 1995 268 R1456 1463 7611522 10.1152/ajpregu.1995.268.6.R1456

[B24] Chen CP Eastwood SL Hope T McDonald B Francis PT Esiri MM Immunocytochemical study of the dorsal and median raphe nuclei in patients with Alzheimer's disease prospectively assessed for behavioural changes Neuropathol Appl Neurobiol 2000 26 347 355 10.1046/j.1365-2990.2000.00254.x 10931368

[B25] Mirmiran M Swaab DF Kok JH Hofman MA Witting W Van Gool WA Circadian rhythms and the suprachiasmatic nucleus in perinatal development, aging and Alzheimer's disease Prog Brain Res 1992 93 151 162 discussion 162-153full_text 1480747 10.1016/s0079-6123(08)64570-7

[B26] Paxinos G Watson C The Rat Brain in Stereotaxic coordinates 1998 New York: Academic press 10.1016/0165-0270(80)90021-7 6110810

[B27] Jinno S Kinukawa N Kosaka T Morphometric multivariate analysis of GABAergic neurons containing calretinin and neuronal nitric oxide synthase in the mouse hippocampus Brain Res 2001 900 195 204 10.1016/S0006-8993(01)02292-2 11334798

[B28] Guzman-Marin R Alam MN Szymusiak R Drucker-Colin R Gong H McGinty D Discharge modulation of rat dorsal raphe neurons during sleep and waking: effects of preoptic/basal forebrain warming Brain Res 2000 875 23 34 10.1016/S0006-8993(00)02561-0 10967295

[B29] McGinty DJ Harper RM Dorsal raphe neurons: depression of firing during sleep in cats Brain Res 1976 101 569 575 10.1016/0006-8993(76)90480-7 1244990

[B30] Jouvet D Vimont P Delorme F Jouvet M Study of selective deprivation of the paradoxal sleep phase in the cat C R Seances Soc Biol Fil 1964 158 756 759 14186938

[B31] Mendelson WB Guthrie RD Frederick G Wyatt RJ The flower pot technique of rapid eye movement (REM) sleep deprivation Pharmacol Biochem Behav 1974 2 553 556 10.1016/0091-3057(74)90018-5 4371007

[B32] Mallick BN Gulyani S Rapid eye movement sleep deprivation increases chloride-sensitive Mg-ATPase activity in the rat brain Pharmacol Biochem Behav 1993 45 359 362 10.1016/0091-3057(93)90251-N 8327542

[B33] Suchecki D Lobo LL Hipolide DC Tufik S Increased ACTH and corticosterone secretion induced by different methods of paradoxical sleep deprivation J Sleep Res 1998 7 276 281 10.1046/j.1365-2869.1998.00122.x 9844854

[B34] De Boer SF Koopmans SJ Slangen JL Van der Gugten J Plasma catecholamine, corticosterone and glucose responses to repeated stress in rats: effect of interstressor interval length Physiol Behav 1990 47 1117 1124 10.1016/0031-9384(90)90361-7 2395915

[B35] Evarts EV Relation of cell size to effects of sleep in pyramidal tract neurons Prog Brain Res 1965 18 81 91 full_text 14329053 10.1016/s0079-6123(08)63585-2

[B36] Kuppermann BD Kasamatsu T Changes in geniculate cell size following brief monocular blockade of retinal activity in kittens Nature 1983 306 465 468 10.1038/306465a0 6646226

[B37] Mallick BN Siegel JM Fahringer H Changes in pontine unit activity with REM sleep deprivation Brain Res 1990 515 94 98 10.1016/0006-8993(90)90581-U 2357583 PMC9045851

[B38] Das G Mallick BN Noradrenaline acting on alpha1-adrenoceptor mediates REM sleep deprivation-induced increased membrane potential in rat brain synaptosomes Neurochem Int 2008 52 734 740 10.1016/j.neuint.2007.09.002 17950953

[B39] McCarty NA O'Neil RG Calcium signaling in cell volume regulation Physiol Rev 1992 72 1037 1061 1332089 10.1152/physrev.1992.72.4.1037

[B40] Urbain N Creamer K Debonnel G Electrophysiological diversity of the dorsal raphe cells across the sleep-wake cycle of the rat J Physiol 2006 573 679 695 10.1113/jphysiol.2006.108514 16613874 PMC1779756

[B41] Descarries L Watkins KC Garcia S Beaudet A The serotonin neurons in nucleus raphe dorsalis of adult rat: a light and electron microscope radioautographic study J Comp Neurol 1982 207 239 254 10.1002/cne.902070305 7107985

[B42] Pal D Mallick BN Neural mechanism of rapid eye movement sleep generation with reference to REM-OFF neurons in locus coeruleus Indian J Med Res 2007 125 721 739 17704548

[B43] Hipolide DC Tufik S Raymond R Nobrega JN Heterogeneous effects of rapid eye movement sleep deprivation on binding to alpha- and beta-adrenergic receptor subtypes in rat brain Neuroscience 1998 86 977 987 10.1016/S0306-4522(98)00067-0 9692733

[B44] Gulyani S Mallick BN Possible mechanism of rapid eye movement sleep deprivation induced increase in Na-K ATPase activity Neuroscience 1995 64 255 260 10.1016/0306-4522(94)00333-Z 7708210

[B45] Majumdar S Faisal M Madan V Mallick BN Increased turnover of Na-K ATPase molecules in rat brain after rapid eye movement sleep deprivation J Neurosci Res 2003 73 870 875 10.1002/jnr.10710 12949914

[B46] Edwards DL Johnston KM Poletti CE Foote WE Morphology of pontomedullary raphe and reticular formation neurons in the brainstem of the cat: an intracellular HRP study J Comp Neurol 1987 256 257 273 10.1002/cne.902560206 3558881

[B47] Groves PM Wilson CJ Fine structure of rat locus coeruleus J Comp Neurol 1980 193 841 852 10.1002/cne.901930402 7430440

[B48] Ramon-Moliner E The locus coeruleus of cat. 3. Light and electron microscopic studies Cell Tissue Res 1974 149 205 221 4608498 10.1007/BF00222274

[B49] Wyllie AH Kerr JF Currie AR Cell death: the significance of apoptosis Int Rev Cytol 1980 68 251 306 10.1016/S0074-7696(08)62312-8 7014501

